# P04.83. What factors influence the use of integrative medicine (IM) modalities by infectious disease (ID) physicians?

**DOI:** 10.1186/1472-6882-12-S1-P353

**Published:** 2012-06-12

**Authors:** K Shere-Wolfe, J Tilburt, C D'Adamo, B Berman, M Chesney

**Affiliations:** 1 University of Maryland, Baltimore, USA; 2 Mayo Clinic, Rochester, USA; 3 University of California, San Franscisco, USA

## Purpose

The purpose was to assess factors that may influence the use of IM modalities by ID physicians in their practice.

## Methods

In a 2010 national survey of 1000 practicing ID physicians, participants were asked to report the extent (major, minor or not at all) to which the following considerations played a role in their recommendation/referral of IM modalities: (1) Knowledge of how and when to use them; (2) Amount of clinical research showing clear benefit; (3) Insurance; (4) Cost; (5) Reliable referral base; (6) Concern for professional reputation; (7) Fear of judgment from colleagues; (8) Insufficient regulatory oversight of supplements; and (9) Potential drug interactions with botanicals/supplements.

## Results

A total of 311 (31%) ID physicians responded to the survey. The mean age was 49 and 64% of respondents were male. Their responses to the questions are listed below.

**Table 1:** 

Factor	Number of respondents	Major role (%)	Minor or Not at all (%)
Drug Interactions	293	82	18

Research	294	80	20

Knowledge	294	72	28

Insurance	292	24	76

Cost	293	39	61

Referral base	288	39	61

Professionl reputation	293	14	86

Fear of judgement	293	4	96

Regulation oversight	294	69	31

## Conclusion

For ID physicians, factors that were considered a major influence on the use of IM modalities included: potential drug interactions, clinical research, knowledge of IM modalities, and regulatory oversight. Factors that played a minor/no role in the use of IM modalities included fear of judgment and concern for professional reputation.

